# Valence Bond Theory Allows a Generalized Description of Hydrogen Bonding

**DOI:** 10.1021/jacs.3c08196

**Published:** 2023-09-04

**Authors:** Sason Shaik, David Danovich, Richard N. Zare

**Affiliations:** † Institute of Chemistry, The Hebrew University of Jerusalem, Jerusalem 9190401, Israel; ‡ Department of Chemistry, Stanford University, Stanford, California 94305, United States

## Abstract

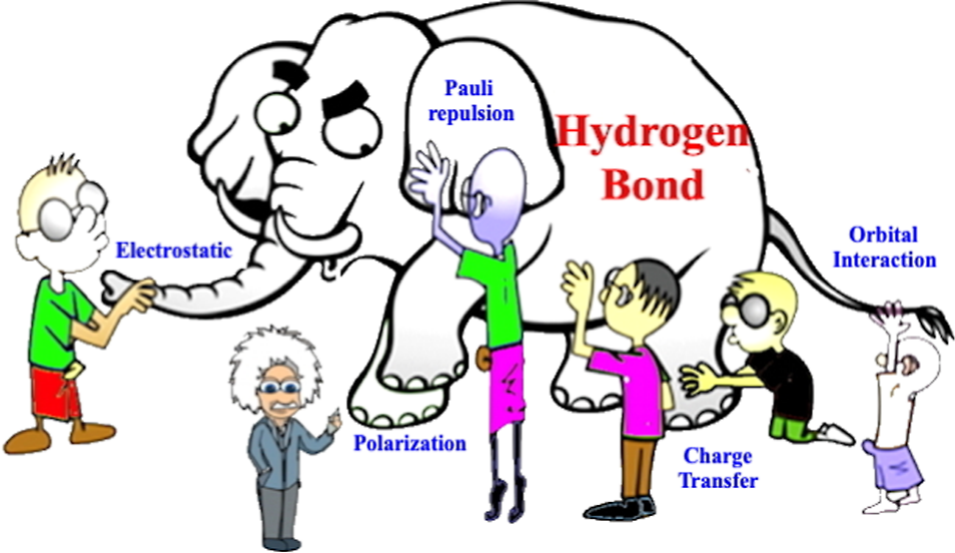

This paper describes the nature of the hydrogen bond (HB), B**:**---H–A, using valence bond theory (VBT). Our analysis shows that the most important HB interactions are polarization and charge transfer, and their corresponding sum displays a pattern that is identical for a variety of energy decomposition analysis (EDA) methods. Furthermore, the sum terms obtained with the different EDA methods correlate linearly with the corresponding VB quantities. The VBT analysis demonstrates that the total covalent-ionic resonance energy (RE_CS_) of the HB portion (B---H in B**:**---H–A) correlates linearly with the dissociation energy of the HB, Δ*E*_diss_. In principle, therefore, RE_CS_(HB) can be determined by experiment. The VBT wavefunction reveals that the contributions of ionic structures to the HB increase the positive charge on the hydrogen of the corresponding external/free O–H bonds in, for example, the water dimer compared with a free water molecule. This increases the electric field of the external O–H bonds of water clusters and contributes to bringing about catalysis of reactions by water droplets and in water-hydrophobic interfaces.

## Introduction

There is a parable of Indian origins about a group of blind men encountering an elephant for the first time.^1,2^ Each person touches a different part of the animal and makes wildly wrong assumptions about what the whole elephant looks like. In many ways, this parable applies to the enigmatic description of the origins of the hydrogen bond (HB).^1^ The HB is often considered to be dominated by electrostatic (ES) interactions, polarization (POL), or covalent interactions in which electrons are shared to make a weak chemical bond. In this study, we show that ionic structures and covalent-ionic resonance energies^3^ make more important contributions than have been previously known. Specifically, we find that using valence bond theory (VBT)^3,4^ considers the HB to be sustained by covalent-ionic resonance energy,^3^ arising from the combined charge transfer and POL effects. This resonance energy is, in principle, available from experiments (see Figure 5). Thus, we are able to present a unified picture of what a HB is. This description covers both strong and weak HBs. The ionic character helps to explain phenomena that have seemed unrelated and somewhat enigmatic; for example, how water in contact with some hydrophobic medium, such as air, oil, or a metal oxide surface, can become “electrified” and drive redox reactions that do not occur in bulk water.^5^

### Brief Review of the Importance and Origins of HBs

**Scheme 1:**
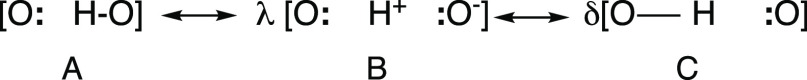
HB between Two Water Molecules; λ and δ Are the Small Contributions of B and C

The existence of HBs was postulated early on,^6^ based on the Lewis theory of the chemical bond.^6b^ Because hydrogen can make only a single bond, Pauling^7a^ initially ruled out any bonding and defined the HB as an ES bond. However, in the 1960 edition of his book,^7b^ he presented HB’s using resonance theory. For example, Pauling described the water dimer in Scheme 1, using resonance structures **A–C**, but estimated **C** (based on the HB distance and the weight of the ionic contribution to the O–H bond) to be marginally 5%.^7b^ However, we show in this study that both structure **C** and the corresponding resonance energy of the HB portion are very significant.

Recognition of the importance of HBs in chemistry has quickly grown. The HB constitutes a key architectural element of matter (e.g., in ice, proteins, and nucleic acids) and it is responsible for the stability of aggregates of small molecules (e.g., heats of vaporization of water and hydrofluoric acid (HF) versus their heavier isoelectronic analogues).^8,9^ HBs also endow matter with special mechanical properties in solution, solids, and polymers.^10^

Indeed, the identification of the interactions that dominate the strength of the HB and of bonds in general has remained a contentious issue and a source of many discussions.^9f,9g,11,12^ Generally speaking, there are two theoretical schools of thought in calculating the strength of HBs.

One school of thought has emerged from the energy decomposition analysis (EDA) schemes of Kitaura–Morokuma,^13^ Ziegler–Rauk,^14^ and Baerends–Bickelhaupt.^15^ EDA methods analyze the HB interaction in terms of ES, POL, Pauli repulsion (EX), charge transfer (CT), and orbital interactions.^15^ By Pauli’s repulsion, it is meant that no two electrons may simultaneously occupy the same quantum state so that when brought together they experience a repulsive interaction.

Commonly used EDA methods emphasize the ES origin of HBs. Similarly, the block-localized wavefunction (BLW) method of Mo et al.,^16^ which is akin to VBT using fragment orbitals,^4^ was used to study relatively weak HBs,^17a^ and the results were compared with those emerging from classical VBT calculations.^17b,17c^ The combined BLW and VBT studies showed that the interaction energies of these weak HBs are dominated by the ES (dipole–dipole) interactions with minor contributions from CT.

The second school of thought is based on the natural bond orbital (NBO) theory,^9e,18,19^ which uses second-order perturbation theory to evaluate the interaction energy of the HBs (B**:** H–A) in terms of the interactions of the donor-occupied orbital *n*_B_ and the acceptor vacant bond orbital, σ_HA_*. The interaction energy is proportional to the square of the Fock (**F**) matrix element and inversely proportional to the NBO orbital energy gap
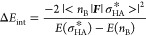
1

As such, NBO theory predicts that the interaction energy (Δ*E*_int_) is dominated by the CT terms from the donor to the acceptor. Δ*E*_int_ in eq 1 is clearly a resonance term owing to the interactions of the orbital on B**:** (*n*_B_) with the antibonding orbital of H–A (σ_HA_*). The related natural EDA (NEDA) method^20^ supports the NBO conclusion that CT is important, but it gives rise to other energy terms, and specifically to a significant ES term.^9g^

The same dichotomy exists for the halogen bond (XB) interaction, where a halogen X atom bridges to two moieties B and A (B**:** X–A). A recent analysis of the XB interaction in terms of BLW and VBT^21^ concluded that in most of the studied XB species, the CT is highly significant, if not the dominant stabilizing term. However, weaker XBs, which were studied by Grabowski,^9g,11^ showed that the POL term is more important than CT. Another concept is the σ-hole, which predicts the linearity of the X-bonded clusters [B---X–Y], caused by the positive charge of the halogen (X) at the tip of its bond axis X–Y.^9g,11,22^ As such, the σ-hole–XY interaction can be considered an ES.

The analysis of total interaction energy into many model terms is bound to create some ambiguities and disagreements (e.g., the effective one-electron operator, the Fock operator in eq 1, includes mono-electronic terms and effective bi-electronic ones). Recently, Pendas et al.^12a^ developed the interacting quantum atoms (IQA) method, which is free of the problematic issues of other EDA methods (e.g., the violation of the Pauli exclusion rule for intermediate wavefunctions used to estimate the EDA energy term). The emerging QM atoms and fragments from the IQA method are well-defined by the quantum theory of atoms in molecules (QTAIM).^23^ Using the interaction in HF---HF as an example,^12a^ Pendas et al. demonstrated that different combinations of the various energy terms can lead to different conclusions about the nature of the HB. Their energy partitions showed that this moderately strong HB may be considered to be dominated by resonance CT or by ES interactions, depending on the groupings of the energy terms. Indeed, Foroutan-Nejad et al.^12b−12d^ demonstrated this path dependency for other bonding interactions. The path dependency obviously poses interpretational ambiguities for EDA.

### VB Outlook of HBs

**Scheme 2:**
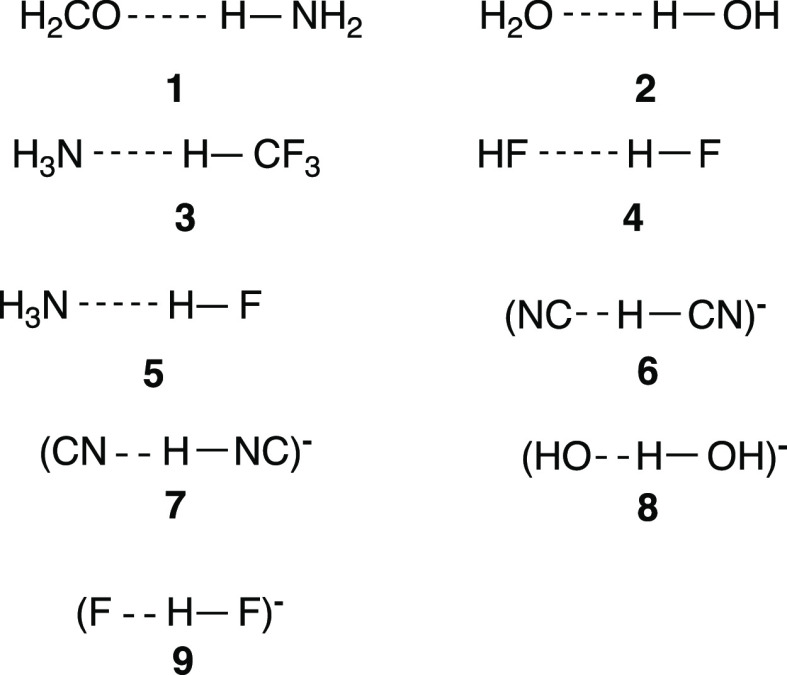
HB Dimers Selected for This Study HBs **6–9** have nearly symmetric geometries.

The present work avoids ambiguities by focusing on the nature of the HB, with the aim of unifying the various approaches and providing a lucid description of the HB in terms of VBT. We selected a series of HBs, which are collected in Scheme 2 and range from weak to strong, such as (FHF)^−^ and (HOHOH)^−^, which are symmetric (or nearly so) HBs^9g,24^ and are most likely to be electronically delocalized species.

The nature of the HB will be determined here by means of classical VBT, which includes covalent and ionic structures. To compare our VB results/conclusions with those from other EDA methods, we studied the various HBs by means of the absolutely localized molecular orbitals (ALMO)-EDA,^25^ NEDA,^20^ and BLW^16^ methods.

A merit of VBT^4^ is that its treatment of the nature of the HB does not use intermediate wavefunctions that violate the Pauli exclusion principle, and at the same time, it uses chemically lucid VB structures. The main character of the HB is the sum of POL and covalent-ionic resonance energy owing to CT and POL. This sum creates a uniform interaction-energy pattern for the nine HBs shown in Scheme 2, and this uniformity is common to a variety of EDA methods (see Figure 3).

The VBT interaction involves chemical structures that flesh out POL and ionic features and provide clearly defined resonance energies that are related to experimental quantities that will be discussed below.^5,26^ Finally, the VB treatment describes the HB as an electron-pair bond,^3^ and we demonstrate that the VB-determined electron-pair bonding of the HB correlates linearly with the dissociation energy of the HB, and as such, the bonding is linked to this observable quantity, which in principle can be determined by experimental means. It is this connection that has not been systematically emphasized in past treatments of this central topic in chemistry and biology. Among better known properties, such as the geometry and strength of the HB, we address the role of covalent-ionic mixing and POL in H-bonded water molecules in water-hydrophobic matter interfaces and water droplet surfaces.^5,26^

## Methods

### Geometry Optimization

The HBs were optimized at the MP2^27^ and CCSD(T)^28^ levels of theory with the cc-pVTZ basis set.^29a^ Details are collected in the Supporting Information.

### VB Calculations

**Scheme 3:**
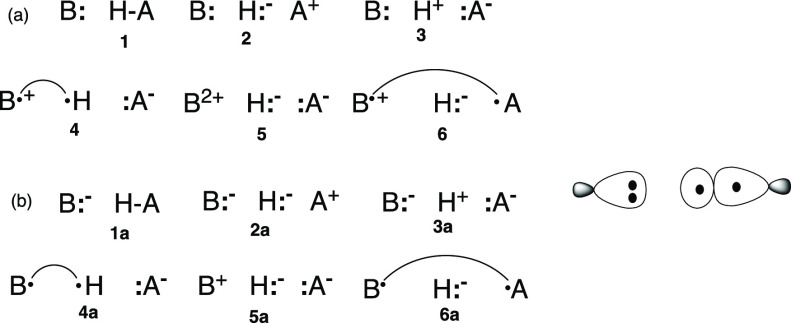
Six VB Structures Used for the VB(6) Level, Which Involves Four Valence Electrons, for B**:** or B**:**^–^ Interacting with H–A; (a) **1–6** for a Neutral B**:**; (b) **1**_**a**_**–6**_**a**_ for an Anionic B**:**^–^; the Arched Lines in Structures **4** (**4**_**a**_) and **6** (**6**_**a**_) Signify Covalent Bond-Coupling of the Respective Two Odd Electrons on B, H, and A; and Drawings of the σ-fragment Orbitals of the HB Are Presented on the Right Side

Classical VB calculations were performed with the XMVB program^30^ using the breathing-orbital VB (L-BOVB) method^31^ and the 6-311G(p,d) basis set.^29b^ We note that the L-BOVB/6-311G(p,d) results for the dissociation energies of the HBs are slightly overestimated vis-à-vis the CCSD(T)/cc-pVTZ quantities. The CCSD(T)/6-311G(p,d) calculated dissociation energies also slightly overestimate the values calculated with CCSD(T)/cc-pVTZ (see Table S10 in the Supporting Information), but the trends in the HB strengths are identical. The following two VB spaces were tested:

(a) VB(6): here the HBs, B**:**-----H–A, were uniformly treated with an active VB space that includes the σ-lone pair of the donor (B**:** or B**:**^–^) and the σ-bond-pair of the H–A bond. This leads to six VB structures, which are displayed in Scheme 3a for cases where B**:** is a neutral molecule (e.g., water and HF), and Scheme 3b where B**:**^–^ is an anion (e.g., F**:**^–^ and HO**:**^–^).
(b) VB(50): the minimal VB(6) active space was tested for HF**:**-----H–F against VB(50), which includes the two electrons of the left-side H–F bond in addition to the 4-electrons used for VB(6) [see Supporting Information for nonzero weighted structures in VB(50)]. The test showed that VB(6) is reasonably accurate.

Note that structures **1–3** (**1**_**a**_**–3**_a_) in Scheme 3 constitute the VB structures that describe the Lewis bond in the H–A molecule [Φ_L_(H–A)]. The structures **3–5** (**3**_**a**_–**5**_a_) in Scheme 3 constitute the electron-pair wavefunction of the B----H bond [Φ_L_(B----H)]. The corresponding wavefunction Φ_L_(B----H) of the HB will account for the total resonance energy of the incipient HB (see Supporting Information). As described above, this quantity varies in correlation with the dissociation energy (Δ*E*_diss_) of the HB and will establish a link between the VB-derived HB description and the experimental quantity that gauges the strength of the HB. Note that structure **6** (**6**_a_) will contribute additional resonance energy, but it was not included in the wavefunction of Φ_L_(B----H) because its contributed resonance energy should be minor owing to the long distance of the paired electrons over B and A.

## Results and Discussion

### Geometries and Charge Distributions of HBs (**1–9**)

**Figure 1:**
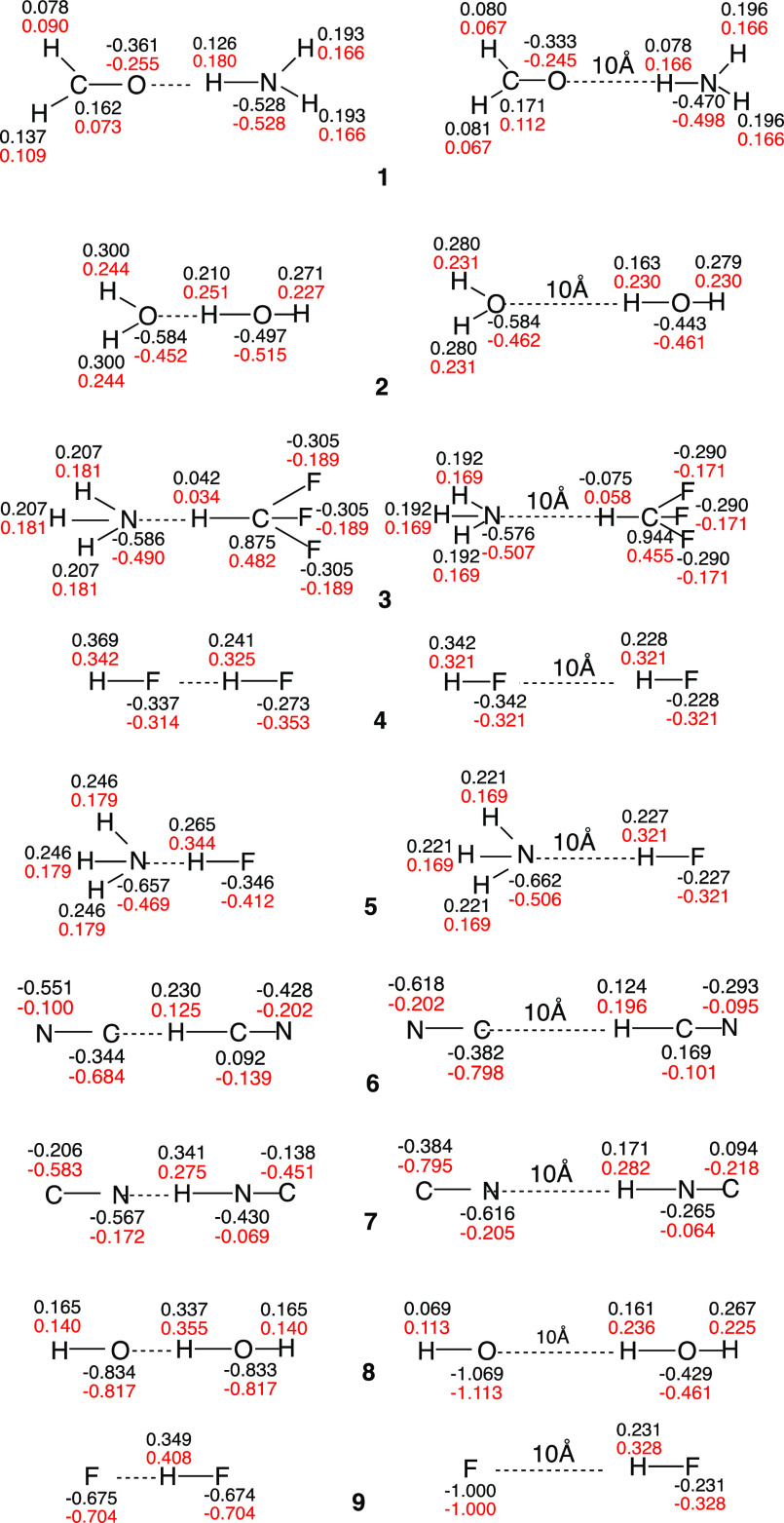
Mulliken charges (BOVB in black and CCSD in red) in the equilibrium geometries of HBs and their dissociated fragments (the distance is 10 Å). Numbers in the bold font near the species correspond to the HB numbers in Scheme 2.

The charges of the optimized HB structures (at the BOVB and CCSD levels) are collected in Figure 1 (the bond lengths and Cartesian coordinates of the optimized HB structures are presented in the Supporting Information). The charges at both levels are often quite similar. The interesting features are that the geometries vary from asymmetric structures, such as the weak HB in the HF**:**-----H–F species, all the way to symmetric ones, such as the anionic species (F---H---F)^−^. In some cases, for example, in the H_2_O dimer (**2**, Figure 1), the hydrogen atoms outside of the HB carry higher positive charges than the Hs in the free molecule (which are represented by the 10 Å distances in Figure 1). The symmetric and rather tight species arise from the dominance of the triple ionic VB structures, for example, F^–^ H^+^ F^–^ in (F---H---F)^−^.^32^ On the other hand, the increased positive charges of the outer Hs, as in the water dimer H_2_O----H–OH, originate in VB structure **4**, which is the covalent structure for the H_2_O----H bonds, and it has a positive charge on H_2_O (B in **4**; Scheme 3a). This positive charge is related to observed redox reactions on the surfaces of water droplets.^5,26e,26f^

### Energy Diagrams of HBs

**Figure 2:**
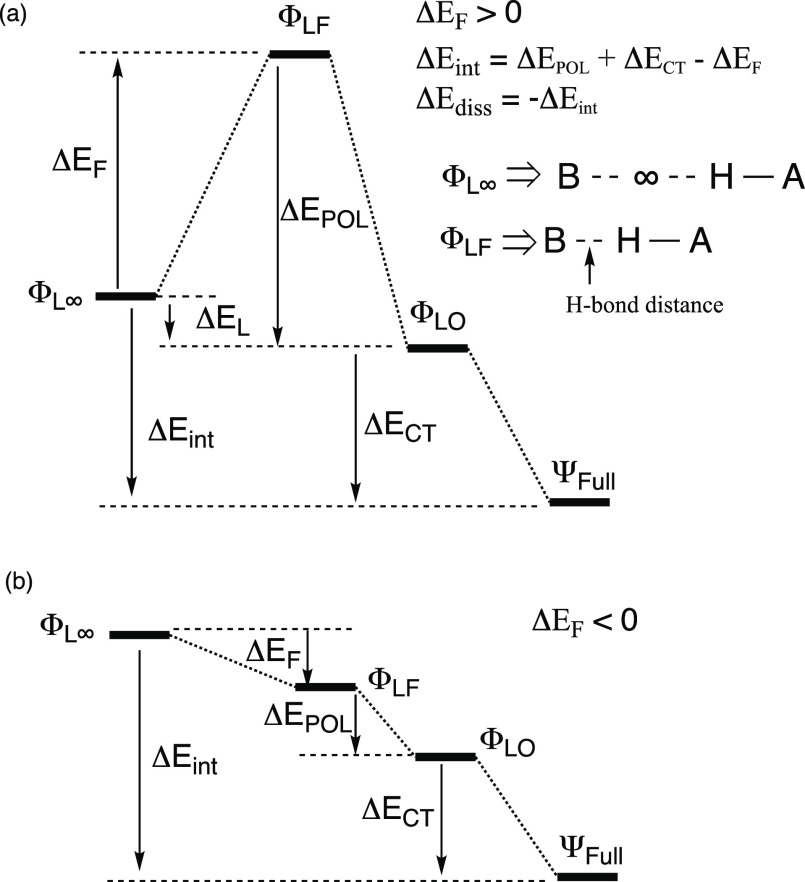
Energy diagrams of HBs with the corresponding energy partition quantities (Δ*E*_F_, Δ*E*_POL_, and Δ*E*_CT_): (a) the diagram for seven HBs (**1**, **4**, and **5–9**). (b) Diagram for H_2_O----HO–H (**2**) and H_3_N----HCF_3_ (**3**). In both (a) and (b), Φ_LO_ is an optimized Lewis state at the HB distance, whereas Ψ_full_ corresponds to the full BOVB wavefunction, allowing mixing of VB structures **4–6**/**4**_**a**_**–6**_**a**_ into Φ_L_.

The energy partition for the HBs in Scheme 2 follows the same philosophy used before for halogen bonds.^21^ As such, we use an economical energy decomposition, which involves the following four steps that are labeled in the energy diagram in Figure 2 and the energy terms in Table 1.

**Table 1:** BOVB/6-311G(p,d) Energy Components (in kcal/mol), Δ*E*_F_, Δ*E*_L_, Δ*E*_POL_, and Δ*E*_CT_, of the HB Energies in **1–9**

HB	Δ*E*_POL_	Δ*E*_CT_	Δ*E*_diss_^a^	Δ*E*_F_	Δ*E*_L_	Δ*E*_CT_ + Δ*E*_POL_
1	–23.16	–1.87	3.14	21.90	–1.27	–25.03
2	–2.23	–1.66	6.00	–2.10	–4.33	–3.89
3	–3.68	–2.61	6.50	–0.21	–3.89	–6.29
4	–9.87	–3.84	7.38	6.32	–3.55	–13.71
5	–15.00	–7.83	15.43	7.40	–7.61	–22.83
6	–19.90	–8.83	21.47	7.26	–12.64	–28.73
7	–61.86	–26.06	29.23	58.68	–3.17	–87.92
8	–72.62	–28.73	37.95	63.40	–9.23	–101.35
9	–60.46	–44.71	58.86	46.30	–14.15	–105.17

a Δ*E*_diss_ was calculated using eq 6. Identical results are obtained from Figure 2.

Initially, we use VB structures **1–3**/**1**_**a**_**–3**_**a**_ (Scheme 3) and calculate the Lewis state, Φ_L∞_, in which the H–A bond is infinitely separated from B**:**. In practice, however, when the distance is 10 Å, the wavefunction does not involve contributions from structures **4–6**/**4**_**a**_**–6**_**a**_ and is considered effective infinite separation (see Supporting Information, Table S17).

We subsequently bring B**:** (B**:**^–^) to the HB distance while freezing the orbitals of the VB wavefunction, which correspond to the base (B**:**/B**:**^–^) and the Lewis bond (H–A), while allowing geometry optimization using the respective VB structures (**1–3** or **1**_**a**_**–3**_**a**_) of the Lewis bond H-A. This generates Φ_LF_, which is the Lewis state at the equilibrium distance of the HB, but with frozen orbitals and Lewis VB structures as in the optimized Lewis state at infinity (10 Å).

The energy change (Figure 2) that accompanies the transformation of the wavefunction from Φ_L∞_ to Φ_LF_ is ΔE_F_. This quantity sums the Pauli repulsion between the H-A molecule and the base (B**:**), the deformation energies of the molecule and the base, and the ES stabilization that arises by bringing these moieties to the HB distance. For most of the HBs we investigated, Δ*E*_F_ > 0 (Figure 2a), but in two cases (H_2_O----HOH, **2**; H_3_N----HCF_3_, **3**) Δ*E*_F_ is negative (Figure 2b). In the latter cases, ES interactions are apparently large enough to overcome the repulsive factors and lead to a negative Δ*E*_F_ quantity.

Subsequently, by optimizing the Lewis structures of Φ_LF_ at the HB distance, we obtain the optimized Lewis state Φ_LO_, where O signifies that the VB structures and orbitals of the Lewis states are optimized for the final HB geometry. This procedure changes the contributions of the individual VB structures within the Lewis wavefunction and brings about the POL of the respective wavefunction, thus resulting in the corresponding energy-lowering Δ*E*_POL_ (Figure 2).

Thereafter, by allowing the mixing of structures **4–6**/**4**_**a**_**–6**_**a**_ into the Lewis state (with further geometry optimization), we obtain the full wavefunction (Ψ_full_), wherein the HB is fully represented. The energy is further lowered by the respective CT mixing energy, Δ*E*_CT_ (Figure 2).

The various energy terms in Figure 2 are as follows
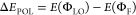
2
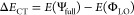
3
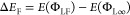
4
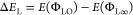
5
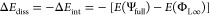
6

Table 1 shows the energy terms for the HBs in Scheme 2. Inspection of the data reveals that, with the exception of H_2_O----HOH and H_2_C=O----HNH_2_ (**2** and **3**), the Δ*E*_F_ quantity is positive and it destabilizes the Lewis state, Φ_LF_. Relative to Φ_LF_, the most important stabilizing factor is the Δ*E*_POL_ term, while Δ*E*_CT_ is a secondary stabilizing effect. Table 1 also shows the sum term Δ*E*_CT_ + Δ*E*_POL_.

### Sum Term (Δ*E*_POL_ + Δ*E*_CT_) Unifies the Trends in the Nine HBs Examined

**Figure 3:**
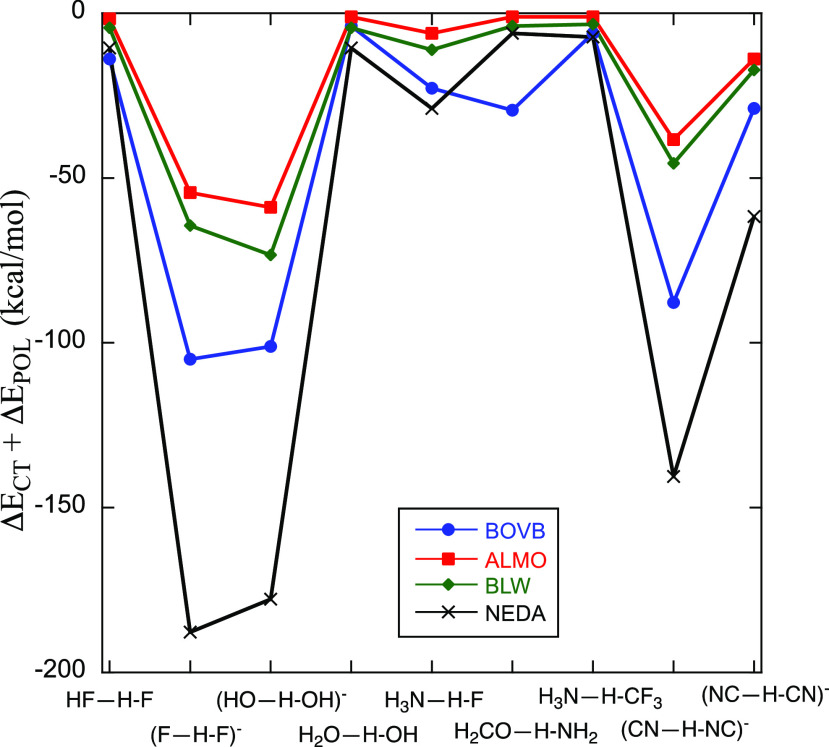
Pattern plot of the sum terms, Δ*E*_CT_ + Δ*E*_POL_, for the family of HBs studied here (specified at the bottom of the plot).

It is important to realize that different methods (e.g., BOVB, ALMO-EDA, NEDA, and BLW) do not fully agree with one another about whether the dominant stabilizing term is Δ*E*_POL_ or Δ*E*_CT_ in a particular HB. By contrast, as shown in Figure 3, all the methods reveal that the sum of the terms, Δ*E*_CT_ + Δ*E*_POL_, exhibits a very similar pattern for the variation of the stabilizing factor across the series of HBs. Thus, Δ*E*_POL_ and Δ*E*_CT_ are entangled, and their relative magnitudes may be path-dependent, but their sum exhibits consistency of patterns for all the HBs studied here. This behavior represents a major advance in our understanding of the nature of the HB.

**Figure 4:**
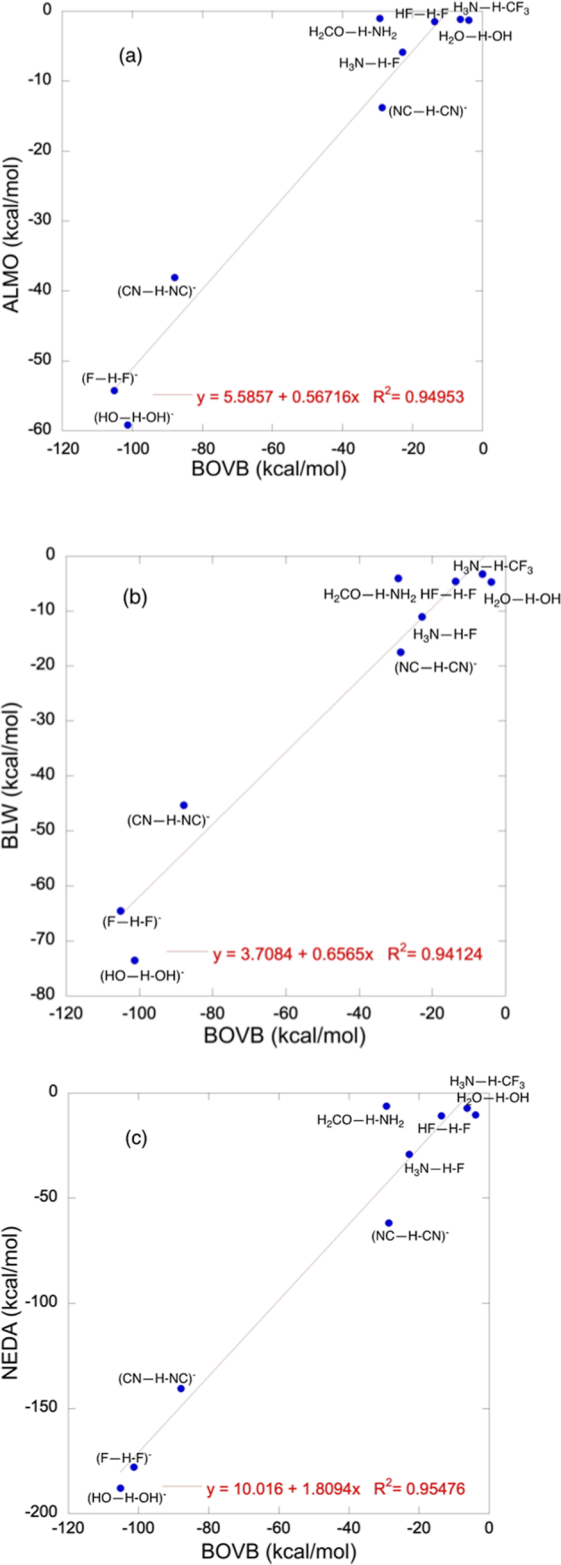
Plots of the sum terms, Δ*E*_POL_ + Δ*E*_CT_, for HBs **1–9**: (a) ALMO versus BOVB. (b) BLW versus BOVB. (c) NEDA versus BOVB.

Furthermore, the sum terms, Δ*E*_CT_ + Δ*E*_POL_, calculated by various methodologies, correlate linearly with one another (see Figure S2 in the Supporting Information). Figure 4 shows that the sum terms, Δ*E*_CT_ + Δ*E*_POL_, calculated with the ALMO-EDA, BLW, and NEDA methods, correlate linearly with the BOVB values with reasonably good correlation coefficients *R*^2^ = 0.94–0.95. Figure S2 in the Supporting Information shows that the methods correlate with one another (ALMO vs NEDA, BLW vs NEDA, and ALMO vs BLW) with almost identical correlation coefficients.

Furthermore, the same sum terms calculated with ALMO^25^ using three different density functional theory (DFT) functionals (ωB97X-D, B3LYP, and ωB97M-rV) agree very closely (see Table S14 in the Supporting Information). As such, it is reasonable to assume that the Δ*E*_POL_ and Δ*E*_CT_ terms in the sum terms are interlinked and represent the major stabilizing factor of the HB.

### Strengths of the HB and Its Relation to the Charge-Shift Resonance Energy

As we described, the mixing of structures **3–5**/**3**_**a**_–**5**_**a**_ gives rise to the resonance energy of the HB moiety B----H in B----H–A. Thus, structure **4**/**4**_**a**_ is the covalent Heitler–London structure of the B----H bond, whereas structures **3**/**3**_**a**_ and **5**/**5**_**a**_ are the corresponding ionic structures. As such, the VB mixing of these three structures at the optimized HB distance (B----H) gives rise to the total charge-shift resonance energy (RE_CS_)^3,33^ of the HB.

Table 2 shows the BOVB calculated RE_CS_ for the nine HBs as well as Δ*E*_diss_ computed by BOVB and CCSD(T) methods. In each case, the RE_CS_ is determined relative to the VB structure of the lowest energy among **3–5**/**3**_**a**_**–5**_**a**_. Thus, for example, for (F---H---F)^−^, the lowest structure is the triple-ion structure **3**_**a**_. The RE_CS_ and Δ*E*_diss_ quantities in Table 2 are not identical, primarily because of the dissociation limit at which the Δ*E*_diss_ value is determined relative to the separated entities, for example, F^–^ + H–F in the case of (F---H---F)^−^, whereas the reference for the RE_CS_ is the lowest VB structure (e.g., the triple-ion F^–^---H^+^---F^–^). Other differences originate in the geometries of the fragments; RE_CS_ is determined by the use of frozen fragment geometries as in the HB, whereas Δ*E*_diss_ uses relaxed fragment geometries.

**Table 2:** RE_CS_ for the B----H Portion of the HBs and the Total Δ*E*_diss_ Values for VB(6)^a^

HB	1	2	3	4	5	6	7	8	9
RE_CS_	3.7	7.2	6.2	6.8	14.3	20.9	41.5	62.8	65.6
Δ*E*_diss_^b^	3.1	6.0	6.5	7.4	15.4	21.5	29.2	38.0	58.9
Δ*E*_diss_^c^	3.1	5.8	4.9	5.3	13.0	22.8	31.9	38.6	54.4
Δ*E*_diss_^d^	3.5	6.7	5.8	5.8	13.9	23.1	33.2	41.7	56.2

a All the energy values are in kcal/mol.

b Corresponds to BOVB/6-311G(p,d).

c Corresponds to CCSD(T)/cc-pVTZ.

d Corresponds to CCSD(T)/6-311G(p,d).

**Figure 5:**
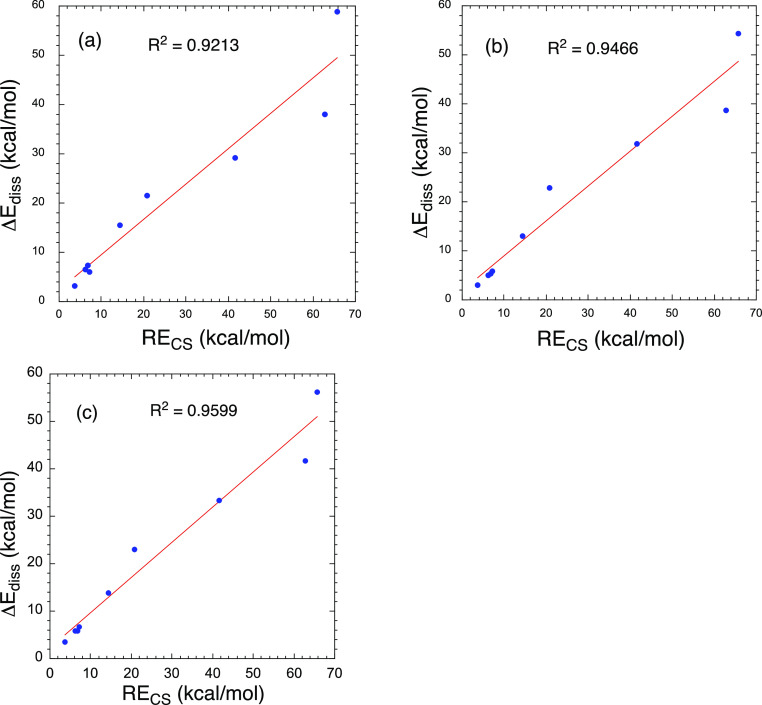
Correlations between RE_CS_ [BOVB/6-311G(p,d)] quantities for the B---H bond of the HB versus Δ*E*_diss_ values of the HBs using (a) BOVB/6-311G(p,d), (b) CCSD(T)/cc-pVTZ, and (c) CCSD(T)/6-311G(p,d). All values are in kcal/mol.

Furthermore, as shown in Figure 5, the RE_CS_ quantities correlate with the dissociation energies (Δ*E*_diss_) of the corresponding HBs (*R*^2^ = 0.92–0.95). In principle, therefore, the VB-determined resonance energy of the HB (RE_CS_) correlates with an experimentally relevant quantity, Δ*E*_diss_, which gauges the thermodynamic stability of the HB. We regard this behavior as another advance in our understanding of the nature of the HB.

### HBs as Drivers of Chemical Reactivity

As described above, some of the HBs in Figure 1 (e.g., the water dimer, **2**, H_3_N–H–CF_3_, **3**, and H_3_N----H–F, **5**) carry larger positive charges on the external H atoms (which are not H-bonded) compared with the charges in the free molecules or those infinitely separated (i.e., 10 Å). This feature arises owing to the contribution of VB structure **4** (e.g., H_2_O^•+^— ^•^H:OH^–^), which makes a significant contribution to the bond makeup of the HB of the water dimer and induces positive charging of the external H’s (the same applies to the other neutral HBs mentioned above).

The charge differences between the external and free Hs are small, but they persist in all of the calculations we used. To further test this phenomenon, we optimized the water tetramer (H_2_O)_4_ and the free H_2_O molecule using the MP2/cc-pVTZ level of theory and found the trend to persist. The resulting NBO/CCSD charges of the external Hs are 0.454 (0.470) versus 0.444 (0.462) in the free molecule; the parenthetical values refer to charges in an aqueous solution. In a water droplet, these external O–H^δ+^ bonds generate electric fields (EFs), which are normal to the surface of the droplet and can lead to unexpected redox reactions.

Recently, Head-Gordon et al.,^26f^ used coarse-grained electron modeling of a water droplet and reported that the fluctuations of the EFs at the surface of the droplet are much larger than the interior of the droplet. When the environmental EF distributions are projected onto the O–H bonds found in the interior versus the surface of a water droplet, the droplet surface EFs of ∼16 MV/cm yield enough power to lower the activation energy to accelerate the rates of chemical reactions by several orders of magnitude.

Zare et al.^5,26e,26g^ showed that water droplets promote a variety of unusual reactions. Thus, water droplets on silica surfaces or in aqueous reactions lead to contact electrification and the generation of H_2_O_2_, which oxidizes organic molecules.^5^ Even when spraying from bulk water, the water droplets lead to the reduction of a variety of organic molecules.^26e^ Other groups^26a−26d,26f^ have expressed similar observations/conclusions.

Clearly, the importance of HBs extends from structural/mechanical effects to the enabling of unusual reactions.

## Conclusions

VBT is used to unify the bonding aspects of the HB in a series of HBs that range from weak to strong ones that are nearly symmetric^24^ [e.g., (HO---H---OH)^−^ and (FHF)^−^].

The two major interactions that make up HBs are due to POL and CT, which are caused by the significant involvement of the ionic VB structures (Scheme 3) in bonding. The sum POL + CT displays the same pattern (displayed in Figure 3), irrespective of which term is actually larger or smaller. Furthermore, the sum terms for VBT correlate linearly (Figure 4) with the corresponding ones obtained from various EDA methods tested in this paper: ALMO (using Hartree-Fock and three different functionals),^25^ BLW,^16,17^ and NEDA.^20^

Additionally, the VB analysis reveals that the RE_CS_ of the B----H portion of the B**:**----HA HB correlates linearly with the dissociation energy of the HB, Δ*E*_diss_ (Figure 5). As such, RE_CS_ values can, in principle, be extracted from plots against experimental Δ*E*_diss_ values.

Finally, in agreement with MP2 and CCSD, VBT reveals that the contributions of ionic structures to the HB increase the positive charge on the hydrogen of the corresponding external O–H bonds in the water dimer, compared with a free water molecule. This increases the EF of the external O–H bonds of water droplets and of water in contact with hydrophobic media, such as air, oil, or a metal oxide surface. In all these cases, the EF of the external O–H bond has been shown to enable chemical reactions not found in bulk water.^5,26^

Thus, HBs are multifaceted. The HB constitutes a key architectural element of ice, proteins, nucleic acids, and many other structures; it is responsible for the stability of aggregates of small molecules;^8,9^ and it endows materials with special mechanical properties in solution, solids, and in soft matter such as proteins and polymers.^10^ Moreover, HBs possess bonding aspects that are more intriguing than isolated bonds in molecules. These bonding mechanisms hold together the HB components by means of significant charge-shift resonance energies, and they electrify Hs in interfaces of water–air/solid/oily matter and endow the HBs with increased reactivity.

## Supporting Information Available

VB diagrams; VB structures; BOVB, MP2, and CCSD(T) computational data; and CCSD(T) calculated *XYZ* coordinates (PDF)

## Dedication

This paper is dedicated to Roald Hoffmann on the occasion of his 85th birthday.

## Abbreviations

BOVB: breathing-orbital valence bond method
VBT: valence bond theory
EDA: energy decomposition analysis
HB: hydrogen bonding
RE: resonance energy
BLW: block-localized wavefunction
NBO: natural bond orbital
ALMO: absolutely localized molecular orbitals
NEDA: natural energy decomposition analyses
EF: electric field
