# Supplementary material for: Valence Bond Theory Allows a Generalized Description of Hydrogen Bonding
Source: J Am Chem Soc. 2023 Sep 4;145(36):20132–40. doi: 10.1021/jacs.3c08196 (PMC10510329; doi:10.1021/jacs.3c08196)
Supplement: Supplementary file 1 — ja3c08196_si_001.pdf [file ja3c08196_si_001.pdf]

Supporting information for

Valence Bond Theory Allows a Generalized Description of Hydrogen Bonding

Sason Shaik*[a(0000-0001-7643-9421)], David Danovich,[a(0000-0002-8730-5119)] Richard N. Zare*[b(0000-0001-5266-4253)]

AFFILIATIONS: [a)] Institute of Chemistry, The Hebrew University of Jerusalem, Jerusalem 9190401, Israel. sason.shaik@gmail.com [b)] Department of Chemistry, Stanford University, Stanford, CA 94305 USA. rnz@stanford.edu

[*)] Authors to whom correspondence should be addressed.

Table of Contents Page

S1. Valence Bond diagrams for 9 Hydrogen-Bonded Molecules that Span the Range from Weak to Strong Bonding using BOVB/6-311G(p,d) calculations 2
Figure S1. Energy Diagrams for HB 1-9 2
S2. The B:----H Portion of the HB (B----H-A) 5
Scheme S1. The three VB structures corresponding to the wavefunction Φ_L(O---H) for HB **1**, H_2CO----H-NH_2. Table S1 5
Scheme S2. The three VB structures corresponding to the wavefunction Φ_L(O---H) for HB **2**, H_2O----H-OH. Table S2 5
Scheme S3. The three VB structures corresponding to the wavefunction Φ_L(N---H) for HB **3**, H_3N----H-CF_3. Table S3 5
Scheme S4. The three VB structures corresponding to the wavefunction Φ_L(F---H) for HB **4**, H-F----H-F. Table S4 6
Scheme S5. The three VB structures corresponding to the wavefunction of Φ_L(N---H) for HB 5, H_3N----H-F. Table S5 6
Scheme S6. The three VB structures corresponding to the wavefunction of Φ_L(C---H) for HB 6, $(NC----H-CN)^-$. Table S6 7
Scheme S7. The three VB structures corresponding to the wavefunction of Φ_L(N---H) for HB **7**, $(CN----H-NC)^-$. Table S7 7
Scheme S8. The three VB structures corresponding to the wavefunction of Φ_L(O---H) for HB **8**, $(HO----H-OH)^-$. Table S8 8
Scheme S9. The three VB structures corresponding to the wavefunction of Φ_L(F---H) for HB 9, $(F----H-F)^-$. Table S9 8
Figure S2. Dependence of the $\Delta E_{CT} + \Delta E_{POL}$ of NEDA vs ALMO (a), BLW vs ALMO (b) and NEDA vs BLW (c) 9
Table S10. BOVB/6-311G(p,d) energies for nonbonded Lewis state ($\Phi_{L\infty}$), frozen nonbonded Lewis state (Φ_{LF}), relaxed Lewis state (Φ_{LO}), relaxed FULL state (Φ_{Full}) which includes charge transfer (CT) structures. Polarization energy (ΔE_{POL}), CT energy (ΔE_{CT}), dissociation energy (ΔE_{Diss}). MP2/cc-pVTZ and CCSD(T)/cc-pVTZ dissociation energies (ΔE_{MP2} and ΔE_{CCSD}). 9

Definition of Energies 10
Table S11. BOVB/6-311G(p,d) polarization energy (ΔE_{POL}), CT energy (ΔE_{CT}), repulsive energy (ΔE_F), Lewis energy (ΔE_L) and dissociation energy (ΔE_{Diss}) 11
Table S12. Total MP2/cc-pVTZ energies for linear and full optimized structures at optimal and long distances. 11
Table S13. ALMO-EDA/HF/6-311G(p,d) energies (kcal/mol) with Hartree-Fock method with BSSE correction 11
Table S14. ALMO-EDA/DFT/6-311G(p,d) energies (kcal/mol) with different DFT methods with BSSE correction 12
Table S15. NEDA/HF/6-311G(p,d) energies (kcal/mol) with Hartree-Fock method (NBO) 12
Table S16. BLW-EDA energies (in kcal/mol) with B3LYP/6-311G(p,d) method 12
Table S17. Changes in dissociation energies $\Delta\Delta E_{diss}$ (kcal/mol) when B-H changes from 10Å to 15Å. Results correspond to BOVB/6-311G(p,d) calculations with 6 structures 13
Table S18. The RE_{CS} and ΔE_{diss} Values (in kcal/mol) for the B----H Portion of the HBs. 13
Figure S3. Correlation between RE_{CS} and ΔE_{diss} calculated for B-H bonds in BOVB/6-311G(p,d) calculations with 3 structures (structures 3-5 or 3a-5a) 13
Figure S4. Mulliken charges in the 4 water molecules cluster for optimized structure (a) and for H-O hydrogen bond at 10.0Å (b). 14
Figure S5. Charges (BOVB in black for 6 structures, in blue for 3 structures (3-5 or 3a-5a) and CCSD in red) 15
S3. The Coulson-Chirgwin weights of the structures from BOVB/6-311G(p,d) calculations with 6 structures 16
S4. Structures with non-zero weights in the 50 structures VBSCF/6-311G(d,p) calculations. 18
S5. Cartesian coordinates (in Å) from CCSD(T)/cc-pVTZ optimized structures 18
References 19

S1. Valence Bond diagrams for 9 Hydrogen-Bonded Molecules that Span the Range from Weak to Strong Bonding using BOVB/6-311G(p,d) calculations.

The diagrams show the energy components (in kcal/mol), ΔE_F, ΔE_{int}, ΔE_{diss}, ΔE_{POL}, and ΔE_{CT} of the HB in molecules **1-9** (see Scheme 2 in the manuscript).

Figure S1: Energy Diagrams for HB 1-9

Molecule 1 H_2CO------H—NH_2

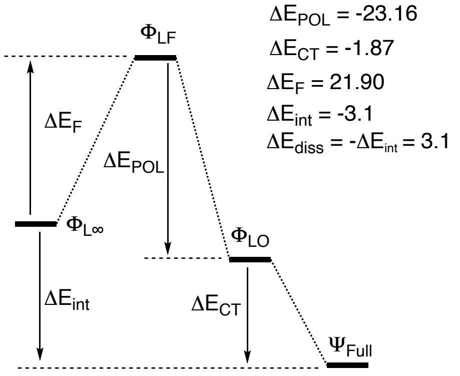

Molecule 2 H_2O-------H—O—H

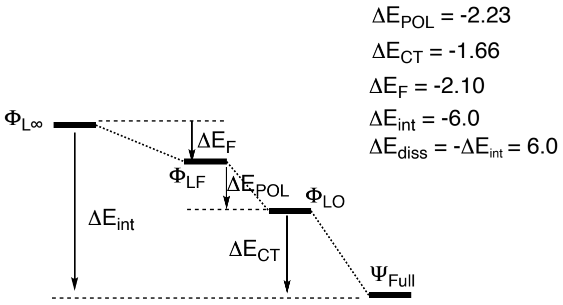

Molecule 3 H_3N-------H—CF_3

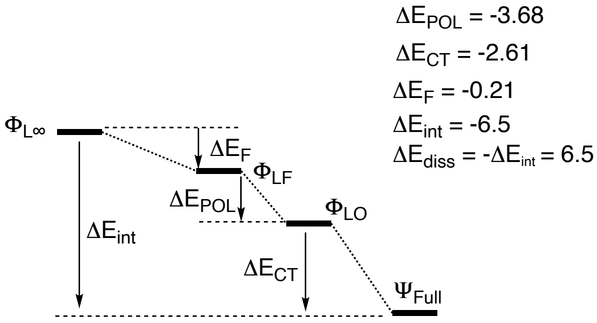

Molecule 4 H—F ----H—F

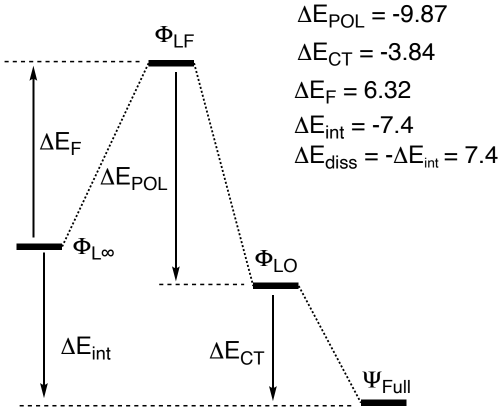

Molecule 5 H_3N-------H—F

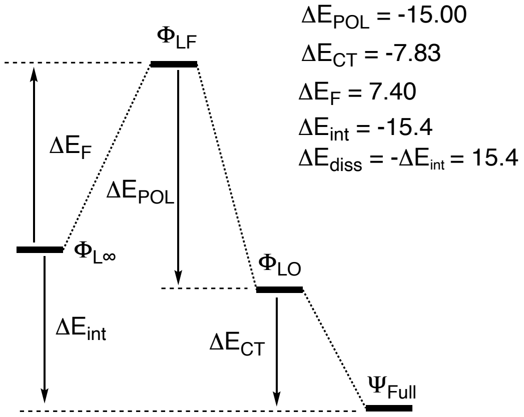

Molecule 6 (NC------H—CN)$^-$

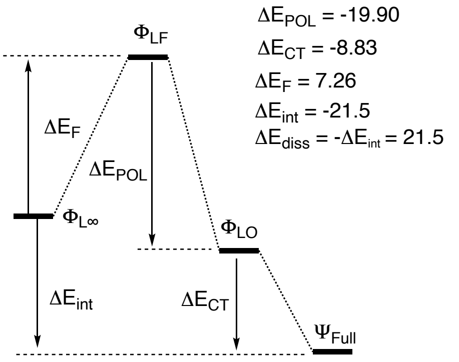

Molecule 7 (CN------H—NC)$^-$

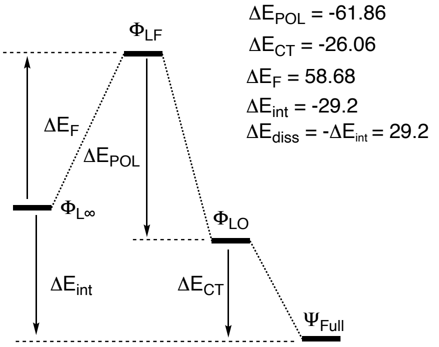

Molecule 8 (H—O-----H –O –H)$^-$

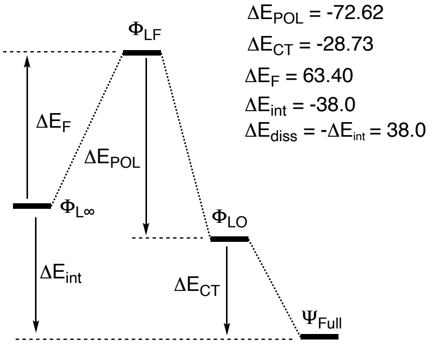

Molecule 9 (F-----H—F)$^-$

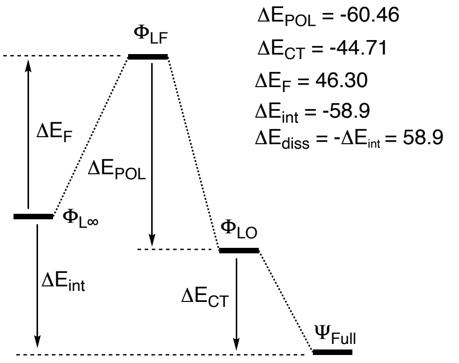

S2. The B:----H Portion of the HB (B----H-A)

The numbers below the structures in Schemes S1-S9 correspond to the numbers of the VB structures in Scheme S3 in the manuscript.

Scheme S1. The three VB structures corresponding to the wavefunction Φ_L(O---H) for HB **1**, H_2CO----H-NH_2.

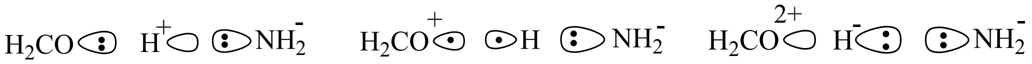

3 4 5

Table S1. Coulson-Chirgwin **w**eights and total energies of the BOVB/6-311G(p,d) calculations at optimal distance (R_{O---H}=2.272 Å) and at 10.0 Å with 3 structures from Scheme S1

	Weights	
	R_{O---H}=2.272 Å	R_{O---H}=10.0 Å
structures		
3	0.960	1.000
4	0.040	0.000
5	~0.000	0.000
E_t	-169.964043	-169.947516

The total energy of the structure 3 (which is the most stable structure) is -169.958147 au at optimal distance (2.272 Å)

Scheme S2. The three VB structures corresponding to the wavefunction Φ_L(O---H) for HB **2**, H_2O----H-OH

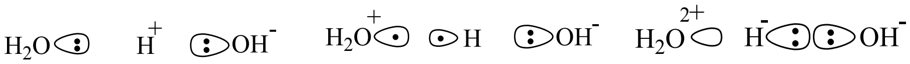

3 4 5

Table S2. Coulson-Chirgwin weights and total energies of the BOVB/6-311G(p,d) calculations at optimal distance (R_{O---H}=1.946 Å) and at 10.0 Å with 3 structures from Scheme S2

	Weights	
	R_{O---H}=1.946 Å	R_{O---H}=10.0 Å
structures		
3	0.937	1.000
4	0.065	0.000
5	~0.000	0.000
E_t	-151.939625	-151.905787

The total energy of the structure 3 (which is the most stable structure) is -151.928170 au at optimal distance (1.946 Å)

Scheme S3. The three VB structures corresponding to the wavefunction Φ_L(N---H) for HB **3**, H_3N----H-CF_3

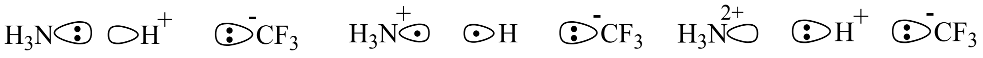

3 4 5

Table S3. Coulson-Chirgwin weights and total energies of the BOVB/6-311G(p,d) calculations at optimal distance (R_{N---H}=2.30 Å) and at 10.0 Å with 3 structures from Scheme S3

	Weights	
	R_{N---H}=2.30 Å	R_{N---H}=10.0 Å
structures		
3	0.936	1.000
4	0.069	0.000
5	~0.000	0.000
E_t	-392.916109	-392.886616

The total energy of the structure **3** (which is the most stable structure) is -392.906215 au at optimal distance (2.30 Å)

Scheme S4. The three VB structures corresponding to the wavefunction Φ_L(F---H) for HB **4**, H-F----H-F

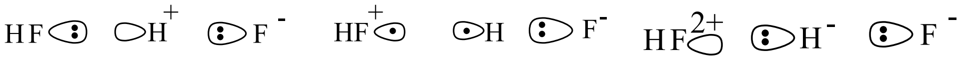

3 **4** **5**

Table S4. Coulson-Chirgwin weights and total energies of the BOVB/6-311G(p,d) calculations at optimal distance (R_{F---H}=1.802 Å) and at 10.0 Å with 3 structures from Scheme S4

	Weights	
	R_{F---H}=1.802 Å	R_{F---H}=10.0 Å
structures		
3	0.948	1.000
4	0.054	0.000
5	~0.000	0.000
E_t	-199.9480819	-199.919980

The total energy of the structure 3 (which is the most stable structure) is -199.9373153 au at optimal distance (1.802 Å)

Scheme S5. The three VB structures corresponding to the wavefunction of Φ_L(N---H) for HB 5, H_3N----H-F

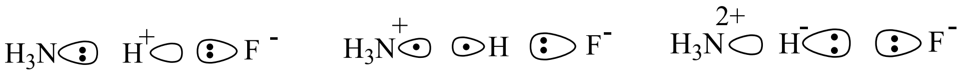

3 **4** **5**

Table S5. Coulson-Chirgwin weights and total energies of the BOVB/6-311G(p,d) calculations at optimal distance (R_{N---H}=1.710 Å) and at 10.0 Å with 3 structures from Scheme S5

	Weights	
	R_{N---H}=1.710 Å	R_{N---H}=10.0 Å
structures		
3	0.875	1.000
4	0.133	0.000
5	~0.000	0.000
E_t	-156.150724	-156.083225

The total energy of the structure 3 (which is the most stable structure) is -156.127868 au at optimal distance (1.710 Å)

Scheme S6. The three VB structures corresponding to the wavefunction of Φ_L(C---H) for HB 6, (NC----H-CN)$^-$

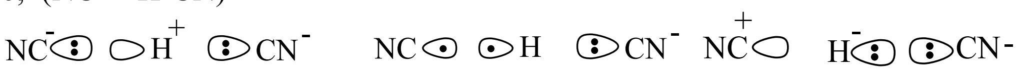

3a **4a** **5a**

Table S6. Coulson-Chirgwin weights and total energies of the BOVB/6-311G(p,d) calculations at optimal distance (R_{C---H}=1.892 Å) and at 10.0 Å with 3 structures from Scheme S6

	Weights	
	R_{C---H}=1.892 Å	R_{C---H}=10.0 Å
structures		
3a	0.868	1.000
4a	0.154	0.000
5a	~0.000	0.000
E_t	-185.155983	-185.075721

The total energy of the structure 3a (which is main structure) is -185.122686 au at optimal distance (1.892 Å)

Scheme S7. The three VB structures corresponding to the wavefunction of Φ_L(N---H) for HB **7**, (CN----H-NC)$^-$

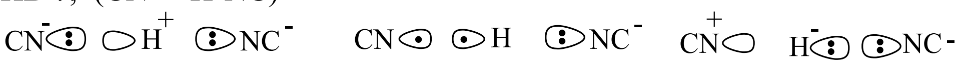

3a **4a** **5a**

Table S7. Coulson-Chirgwin weights and total energies of the BOVB/6-311G(p,d) calculations at optimal distance (R_{N---H}=1.383 Å) and at 10.0 Å with 3 structures from Scheme S7

	Weights	
	R_{N---H}=1.383 Å	R_{N---H}=10.0 Å
structures		
3a	0.716	1.000
4a	0.314	0.000
5a	~0.000	0.000
E_t	-185.201289	-185.066697

The total energy of the structure 3a (which is main structure) is -185.135127 au at optimal distance (1.383 Å)

Scheme S8. The three VB structures corresponding to the wavefunction of Φ_L(O---H) for HB **8**, (HO----H-OH)$^-$

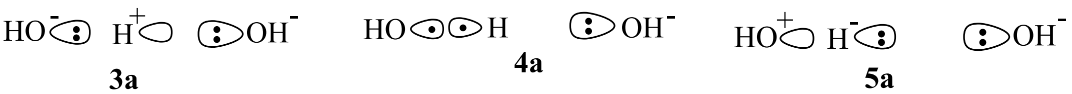

3a **4a** **5a**

Table S8. Coulson-Chirgwin weights and total energies of the BOVB/6-311G(p,d) calculations at optimal distance (R_{O---H}=1.218 Å) and at 10.0 Å with 3 structures from Scheme S8

	Weights	
	R_{O---H}=1.218 Å	R_{O---H}=10.0 Å
structures		
3a	0.585	1.000
4a	0.451	0.000
5a	~0.000	0.000
E_t	-151.428788	-151.225585

The total energy of the structure 3a (which is main structure) is -151.328757 au at optimal distance (1.218 Å)

Scheme S9. The three VB structures corresponding to the wavefunction of Φ_L(F---H) for HB 9, $(F----H-F)^-$

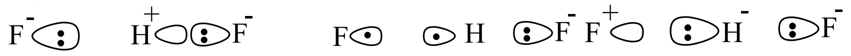

3a **4a** **5a**

Table S9. Coulson-Chirgwin weights and total energies of the BOVB/6-311G(p,d) calculations at optimal distance (R_{F---H}=1.137 Å) and at 10.0 Å with 3 structures from Scheme S9

	Weights	
	R_{F---H}=1.137 Å	R_{F---H}=10.0 Å
structures	Coulson	Coulson
3a	0.600	1.000
4a	0.431	0.000
5a	~0.000	0.000
E_t	-199.525170	-199.291022

The total energy of the structure 3a (which is main structure) is -199.420587 au at optimal distance (1.137 Å)

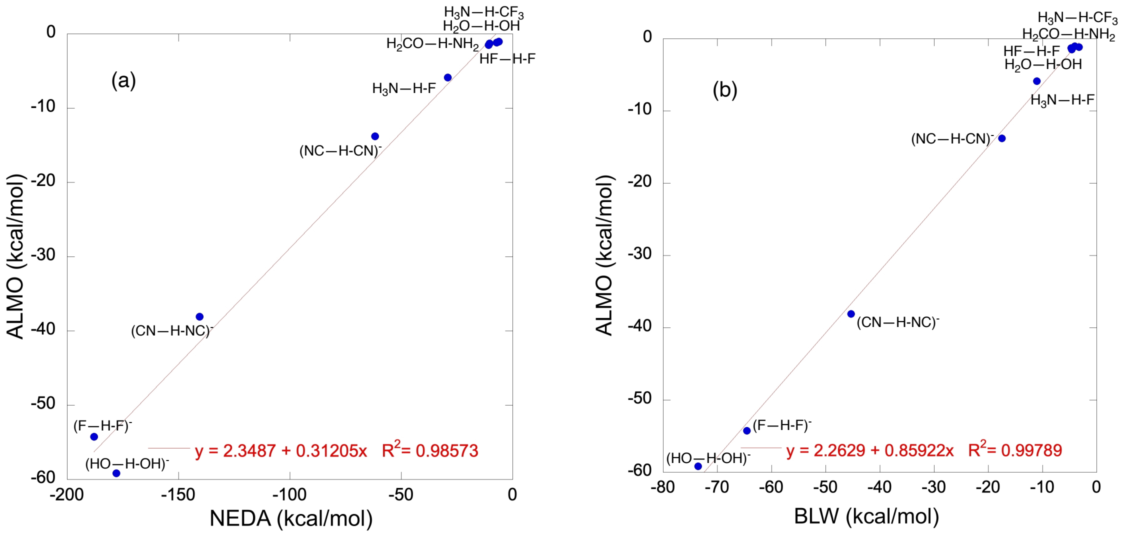

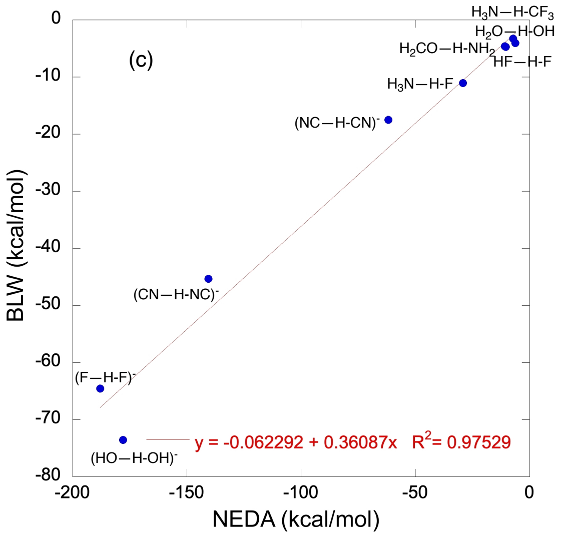

Figure S2. Dependence of the $\Delta E_{CT} + \Delta E_{POL}$ of NEDA vs ALMO (a), BLW vs ALMO (b) and NEDA vs BLW (c)

Table S10. BOVB/6-311G(p,d) energies for nonbonded Lewis state ($\Phi_{L\infty}$), frozen nonbonded Lewis state (Φ_{LF}), relaxed Lewis state (Φ_{LO}), relaxed FULL state (Φ_{Full}) which includes charge transfer (CT) structures. Polarization energy (ΔE_{POL}), CT energy (ΔE_{CT}), dissociation energy (ΔE_{Diss}). MP2/cc-pVTZ and CCSD(T)/cc-pVTZ dissociation energies (ΔE_{MP2} and ΔE_{CCSD}). ΔE values are in kcal/mol, and Φ values in au.

HB	$\Phi_{L\infty}$	Φ_{LF}	Φ_{LO}	Φ_{Full}	ΔE_{POL}	ΔE_{CT}	ΔE_{Diss}	ΔE_{MP2}	$\Delta E_{CCSD(T)}$[j]
1[a]	-.127690	-.092795	-.129708	-.132694	-23.16	-1.87	3.14	3.17	3.08 (3.53)
2[b]	-.094409	-.097754	-.101308	-.103945	-2.23	-1.66	6.00	6.00	5.83 (6.70)
3[c]	-.091833	-.092167	-.098031	-.102202	-3.68	-2.61	6.50	5.02	4.92 (5.79)
4[d]	-.110194	-.100123	-.115844	-.121955	-9.87	-3.84	7.38[k]	5.35	5.27 (5.79)
5[e]	-.273483	-.261698	-.285609	-.298082	-15.00	-7.83	15.43	13.71	12.98 (13.85)
6[f]	-.231573	-.220009	-.251721	-.265790	-19.90	-8.83	21.47	23.32	22.80 (23.05)
7[g]	-.219830	-.126311	-.224886	-.266418	-61.86	-26.07	29.23	33.72	31.91 (33.25)
8[h]	-.414143	-.313114	-.428846	-.474631	-72.62	-28.73	37.95	39.77	38.60 (41.68)
9[i]	-.479647	-.405859	-.502202	-.573453	-60.46	-44.71	58.86	54.88	54.40 (56.23)

a) -170.0 au, b) -152.0 au, c) -393.0 au, d) -200.0 au, e) -155.0 au, f) -185.0 au, g) –185.0 au, h) -151.0 au, i) -199.0 au, j) Results of CCSD(T)/6-311G(p,d) in parenthesizes, k) At the VBSCF level VB(6) = 4.81 kcal/mol, while VB(50) = 3.66 kcal/mol.

Structures used for $\Phi_{L\infty}$ (distance between F and H is 10 Å). $\Phi_{L\infty}$ is a Lewis state, with the "electron donor (or base)", with optimized orbitals at 10Å distance

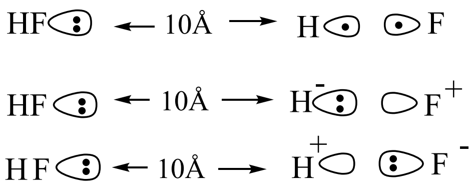

Structures used for Φ_{LO} (distance between F and H is optimal). Φ_{LO} is a Lewis state with optimized orbitals at the equilibrium distance of the hydrogen bond.

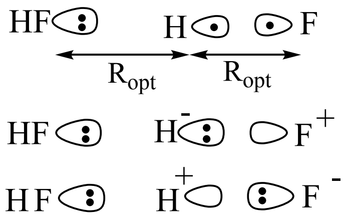

Structures used for Φ_{LF} (distance between F and H is optimal but wavefunction (orbitals) is taken from the $\Phi_{L\infty}$ calculation and is not reoptimized). Φ_{LF} is Frozen Lewis state with optimized orbitals from the long-distance calculation $\Phi_{L\infty}$, calculated at the equilibrium distance without allowing the orbitals to relax. Φ_{LF} rises from Pauli repulsion and the deformation energy of the fragments and stabilized by the electrostatic interaction.

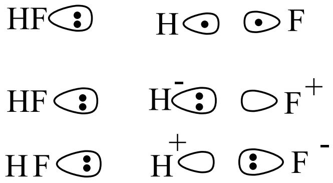

Structures used for Ψ_{Full} (distance between F and H is optimal). Ψ_{Full} is a state that includes optimized orbitals from the Lewis structure and charge transfer structures at the equilibrium distance

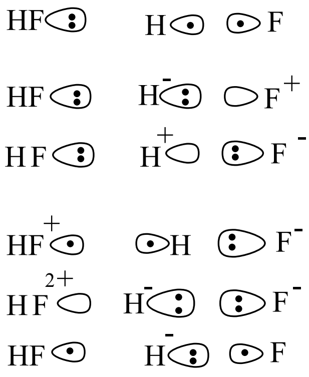

Definition of Energies

$\Delta E_{POL} = E(\Phi_{LO}) - E(\Phi_{LF})$
$\Delta E_{CT} = E(\Psi_{Full}) - E(\Phi_{LO})$
$\Delta E_{F} = E(\Phi_{LF}) - E(\Phi_{L\infty})$
$\Delta E_{L} = E(\Phi_{LO}) - E(\Phi_{L\infty})$
$\Delta E_{int} = E(\Psi_{Full}) - E(\Phi_{L\infty})$
$\Delta E_{diss} = E(\Phi_{L\infty}) – E(\Psi_{Full})$
$\Delta E_{diss} = -\Delta E_{int}$

Φ_{LO} is a Lewis state with optimized orbitals at the equilibrium distance
Φ_{LF} is a Lewis state with optimized orbitals from the long distance calculated at the equilibrium distance without allowing the orbitals to relax

$\Phi_{L\infty}$ is a state with optimized orbitals at long distance
Ψ_{Full} is a state that includes optimized orbitals from the Lewis structure and charge transfer structures at the equilibrium distance

Table S11. BOVB/6-311G(p,d) polarization energy (ΔE_{POL}), CT energy (ΔE_{CT}), repulsive energy (ΔE_F), Lewis energy (ΔE_L) and dissociation energy (ΔE_{diss}) (in kcal/mol)

**HB**	ΔE_{POL}	ΔE_{CT}	$\Delta E_{diss}{}^{a}$	ΔE_F	ΔE_L	ΔE_{CT} + ΔE_{POL}
1	-23.16	-1.87	3.14 (3.13)	21.90	-1.27	-25.03
2	-2.23	-1.66	6.00 (5.99)	-2.10	-4.33	-3.89
3	-3.68	-2.61	6.50 (6.50)	-0.21	-3.89	-6.29
4	-9.87	-3.84	7.38 (7.39)	6.32	-3.55	-13.71
5	-15.00	-7.83	15.43 (15.43)	7.40	-7.61	-22.83
6	-19.90	-8.83	21.47 (21.47)	7.26	-12.64	-28.73
7	-61.86	-26.06	29.23 (29.24)	58.68	-3.17	-87.92
8	-72.62	-28.73	37.95(37.95)	63.40	-9.23	-101.35
9	-60.46	-44.71	58.86 (58.87)	46.30	-14.15	-105.17

a) ΔE_{diss} was calculated as a difference in the total energies of Ψ_{Full} and $\Phi_{L\infty}$. In the parentheses ΔE_{diss} was calculated using equation $\Delta E_{diss} = \Delta E_{POL} + \Delta E_{CT} - \Delta E_F$.

Table S12. Total MP2/cc-pVTZ energies for linear and full optimized structures at optimal and long distances. At long distances (10Å) only bond lengths were reoptimized while angles were kept as in the optimal distance. ΔE_t(opt) is the energy difference between total energies at optimal distances for linear and fully optimized structures. ΔE_{Lin} and ΔE_{Full} are the energy differences between the structures at optimal and long distances. is the energy differences between the linear structures at optimal and long distances calculated at the CCSD(T)/cc-pVTZ level. ΔE_t(opt)= E_t(opt, linear) - E_t(opt,ful), ΔE_{Lin}= E_t(opt,linear) - E_t(long,linear), ΔE_{Full}= E_t(opt,full) - E_t(long,full). E_t in au, ΔE_t in kcal/mol

	Linear		Full					
**HB**	E_t(opt)	E_t(long)	E_t(opt)	E_t(long)	ΔE_t(opt)	ΔE_{Lin}	ΔE_{Full}	$\Delta E_{Lin\text{-}CCSD(T)}$
1[a]	-.765290[j]	-.760236	-.767565[0]	-.760181	1.43	-3.17	4.63	3.08
2[b]	-.646964[0]	-.637403	-.647012[0]	-.637399	0.03	-6.00	6.03	5.83
3[c]	-.280010[0]	-.272013	-.280010[0]	-.272013	0.00	-5.02	5.02	4.92
4[d]	-.668159[0]	-.659639	-.668343[0]	-.659639	0.12	-5.35	5.46	5.27
5[e]	-.804200[0]	-.782357	-.804200[0]	-.782357	0.0	-13.71	13.71	12.98
6[f]	-.966914[0]	-.929749	-.966914[0]	-.929749	0.00	-23.32	23.32	22.80
7[g]	-.955070[0]	-.901327	-.955070[0]	-.901327	0.00	-33.72	33.72	31.91
8[h]	-1.030594[0]	-.967220	-1.030767[0]	-.968211	0.11	-39.77	39.25	38.60
9[i]	-.112551[0]	-.025096	-.112551[0]	-.025096	0.00	-54.88	54.88	54.40

a) -170.0 au, b) -152.0 au, c) -394.0 au, d) -200.0 au, e) -156.0 au, f) -185.0 au, g) -185.0 au, h) -151.0 au, i) -200.0 au j) Imaginary frequency of molecule 6 is -57.41 with linear restriction; all other molecules with linear restriction have no imaginary frequencies.

Table S13. ALMO-EDA/HF/6-311G(p,d) energies (kcal/mol) with the Hartree-Fock method including BSSE correction

**HB**	ΔE_{POL}	ΔE_{CT}	ΔE_{int}	ΔE_{CT} + ΔE_{POL}
1	-0.62	-0.42	-2.30	-1.04
2	-0.89	-0.40	-4.03	-1.29
3	-0.80	-0.37	-4.31	-1.17

4	–0.69	–0.73	–3.99	–1.48
5	-2.95	-2.88	-10.75	-5.83
6	-7.49	-6.29	-31.33	-13.78
7	-18.62	-19.47	-40.37	-38.09
8	-33.92	-25.23	-52.88	-59.15
9	-25.93	-28.32	-73.57	-54.25

Table S14. ALMO-EDA/DFT/6-311G(p,d) energies (kcal/mol) with different DFT methods with BSSE correction

**HB**	ωB97X-D			B3LYP			ωB97M-rV		
	ΔE_{POL}	ΔE_{CT}	ΔE_{int}	ΔE_{POL}	ΔE_{CT}	ΔE_{int}	ΔE_{POL}	ΔE_{CT}	ΔE_{int}
1	-0.49	-1.11	-3.99	-0.49	-1.14	-2.79	-0.49	-0.81	-4.03
2	-0.74	-1.31	-5.45	-0.78	-1.34	-4.95	-0.78	-0.94	-5.35
3	-0.68	-0.94	-5.46	-0.69	-1.04	-4.53	-0.69	-0.75	-5.16
4	–0.59	–1.88	–4.68	-0.62	-1.89	-4.83	-0.62	-1.51	-4.97
5	-2.64	-4.52	-13.01	-2.73	-4.63	-12.29	-2.79	-3.76	-12.26
6	-7.49	-8.77	-35.99	-7.66	-9.35	-35.77	-7.57	-7.98	-35.51
7	-17.73	-22.57	-44.49	-18.04	-23.11	-43.65	-18.19	-21.36	-43.98
8	-29.86	-27.22	-53.27	-30.22	-27.04	-51.37	-30.66	-25.46	-52.25
9	-23.44	-31.72	-72.01	-23.87	-31.16	-70.59	-24.08	-29.87	-70.97

Table S15. NEDA/HF/6-311G(p,d) energies (kcal/mol) with the Hartree-Fock method (NBO)

**HB**	ΔE_{ELECT}	ΔE_{POL}	ΔE_{CT}	ΔE_{int}	$\Delta E_{CT} + \Delta E_{POL}$
1	-5.33	-2.00	-4.30	-1.38	-6.30
2	-10.57	-2.46	-7.95	-4.03	-10.41
3	-9.36	-2.52	-4.63	-4.30	-7.15
4	–8.56	–1.74	–9.05	–3.98	–10.79
5	-22.82	-0.29	-28.84	-10.75	-29.13
6	-39.49	-9.74	-52.15	-21.13	-61.89
7	-63.16	-18.00	-122.59	-40.36	-140.59
8	-86.11	-13.97	-163.94	-52.87	-177.91
9	-94.37	-10.08	-177.88	-73.88	-187.96

$\Delta E_{ELECT} = \Delta E_{POL} + \Delta E_{ES} + \Delta E_{SE}$

Table S16. BLW-EDA energies (in kcal/mol) with the B3LYP/6-311G(p,d) method

**HB**	ΔE_b	ΔE_{int}	ΔE_{CT}	ΔE_{POL}	$\Delta E_{CT} + \Delta E_{POL}$
1	-5.23	-5.14	3.60	0.49	4.09
2	-7.51	-7.51	3.93	0.78	4.71
3	-5.76	-5.65	2.59	0.69	3.28
4	-6.89	-6.89	3.99	0.62	4.61
5	-15.94	-15.76	8.33	2.73	11.06
6	-27.67	-26.11	10.93	6.55	17.48
7	-47.56	-35.89	27.05	18.29	45.34
8	-67.52	-45.25	43.33	30.22	73.55
9	-80.68	-60.64	40.77	23.79	64.56

Definition of the energy terms in Table 16

$\Delta E_b = (E_A + E_B) + BSSE + \Delta E_{disp} - E_{MO}$

$\Delta E_{int} = \Delta E_b - \Delta E_{Def}$ (where $\Delta E_{Def} = (E_A - E_{A\text{-}opt}) + (E_B - E_{B\text{-}opt})$)

$\Delta E_{CT} = E_{MO} - E_{BLW} + BSSE$ calculated at the optimal geometry of the complex

$\Delta E_{POL} = E_{BLW} - E_{BLW0}$

Table S17. Changes in the dissociation energies $\Delta\Delta E_{diss}$ (kcal/mol) upon changing the B-H distance from 10 Å to 15 Å. `the results correspond to BOVB/6-311G(p,d) calculations with VB(6)

**HB**	1	2	3	4	5	6	7	8	9
$\Delta\Delta E_{diss}$				0.35	0.38	1.23	1.13		1.33

Table S18. The RE_{CS} and ΔE_{diss} Values (in kcal/mol) for the B----H Portion of the HBs. The results correspond to BOVB/6-311G(p,d) calculations with 3 structures (structures **3-5** or **3a-5a**) from Scheme 3 in the manuscript.

**HB**	**1**	**2**	**3**	**4**	**5**	**6**	**7**	**8**	**9**
RE_{CS}	3.70	7.19	6.21	6.76	14.34	20.89	41.52	62.77	65.63
ΔE_{diss}	10.37	21.23	18.51	17.63	42.36	50.37	84.46	127.51	146.93

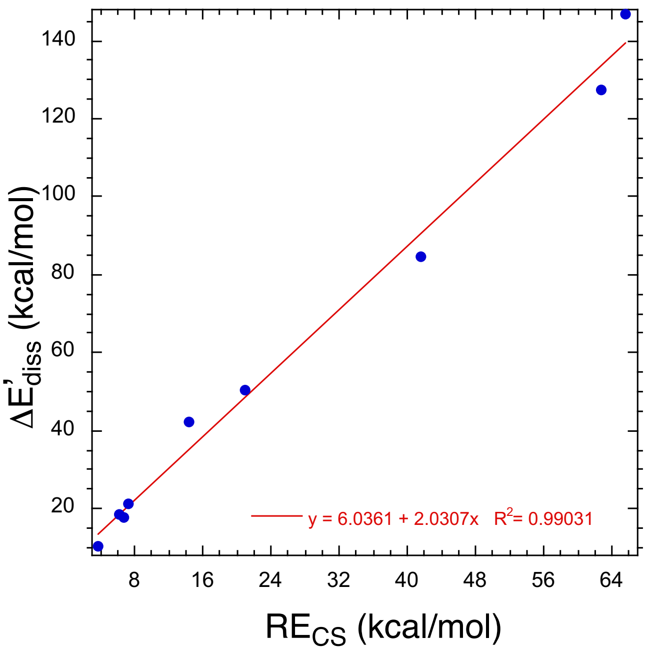

Figure S3. The correlation between RE_{CS} and $\Delta E'_{diss}$ values, calculated for the B---H bond portions in BOVB/6-311G(p,d) calculations with 3 structures (structures **3-5** or **3a-5a**) from Scheme 3 in the manuscript. Note that the dissociation of this bond is computed with the three structures which constitute the bond (**3-5** or **3a-5a**). Thus, $\Delta E'_{diss}$ is different than the ΔE_{diss} values for the entire VB(6) set.

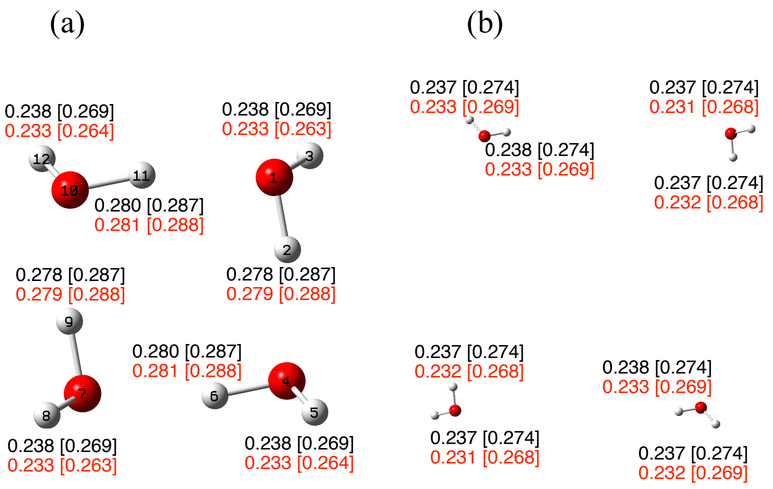

Figure S4. Mulliken charges in the $(H_2O)_4$ cluster, at the corresponding optimized structure (a) and for H-O hydrogen bond at 10.0 Å (b). The first line (black color) corresponds to MP2/cc-pVTZ charges in the gas-phase. Numbers in square parenthesizes correspond to MP2/cc-pVTZ charges in water solution. The second line (red color) corresponds to CCSD/cc-pVTZ charges in the gas-phase. Numbers in square parenthesizes correspond to CCSD/cc-pVTZ in water solution.

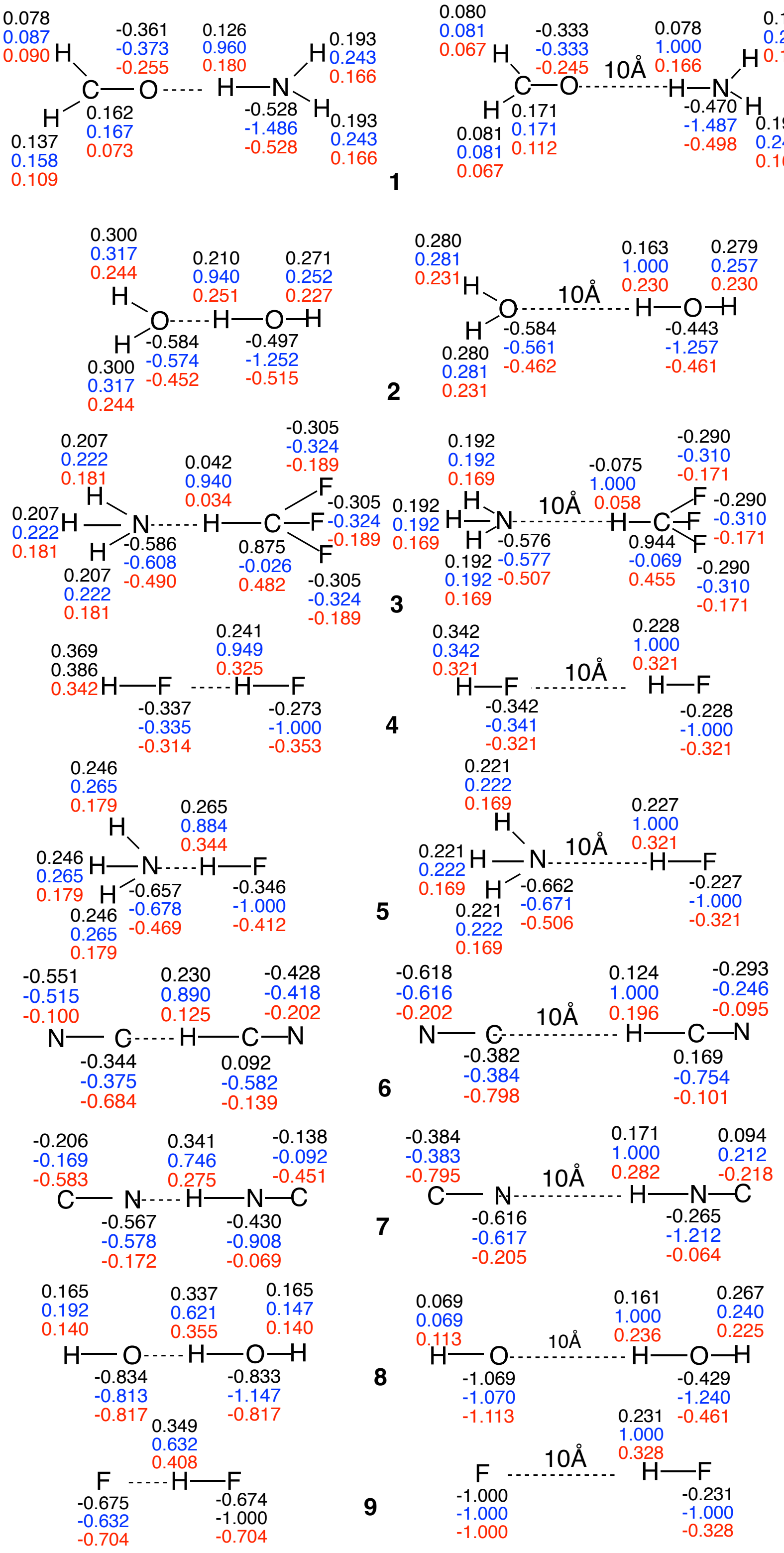

Figure S5. Mulliken charges in the equilibrium geometries of HBs and their dissociated fragments (the distance is 10 Å). Numbers in black correspond to the BOVB/6-311G(p,d) calculations with 6 VB structures in Scheme 3 in the manuscript. Numbers in blue correspond to the BOVB/6-311G(p,d) calculations with 3 structures in Schemes S1– S9 which describe B-H portion of HB (B----H-A). Numbers in red correspond to CCSD/cc-pVTZ calculations. The numbers in the bold font near the species correspond to the HB numbers in Scheme 2 in the manuscript.

S3. The Coulson-Chirgwin weights of the six VB structures (see Scheme 2 in the manuscript) at the BOVB/6-311G(p,d) level. (in parenthesizes, bond length of the corresponding hydrogen bond)

HB 1 (R_{O---H}=2.272 Å)

	Weights
Structures	
1	0.652
2	0.102
3	0.230
4	0.014
5	~0.000
6	0.002

HB 2 (R_{O---H}=1.946 Å)

	Weights
Structures	
1	0.600
2	0.080
3	0.305
4	0.000
5	~0.000
6	0.016

HB 3 (R_{N---H}=2.300 Å)

	Weights
Structures	
1	0.687
2	0.136
3	0.144
4	0.033
5	~0.000
6	~0.000

HB 4 (R_{F---H}=1.802 Å)

	Weights
Structures	
1	0.572
2	0.076
3	0.320
4	0.029
5	~0.000
6	0.004

HB 5 (R_{N---H}=1.710 Å)

	Weights
Structures	

1	0.532
2	0.057
3	0.330
4	0.073
5	~0.000
6	0.007

HB 6 (R_{C---H}=1.892 Å)

	Weights
Structures	
1a	0.615
2a	0.025
3a	0.255
4a	0.105
5a	~0.000
6a	~0.000

HB 7 (R_{N---H}=1.383 Å)

	Weights
Structures	
1a	0.432
2a	0.000
3a	0.341
4a	0.227
5a	~0.000
6a	~0.000

HB 8 (R_{O---H}=1.218 Å)

	Weights
Structures	
1a	0.307
2a	0.000
3a	0.362
4a	0.307
5a	~0.000
6a	0.024

HB 9 (R_{F---H}=1.137 Å)

	Weights
Structures	
1a	0.304
2a	0.000
3a	0.370
4a	0.305
5a	~0.000
6a	0.021

S4. VB Structures with non-zero weights in the 50 structures VBSCF/6-311G(d,p) calculations. The numbers below the structure are Coulson-Chirgwin weights of the corresponding structures

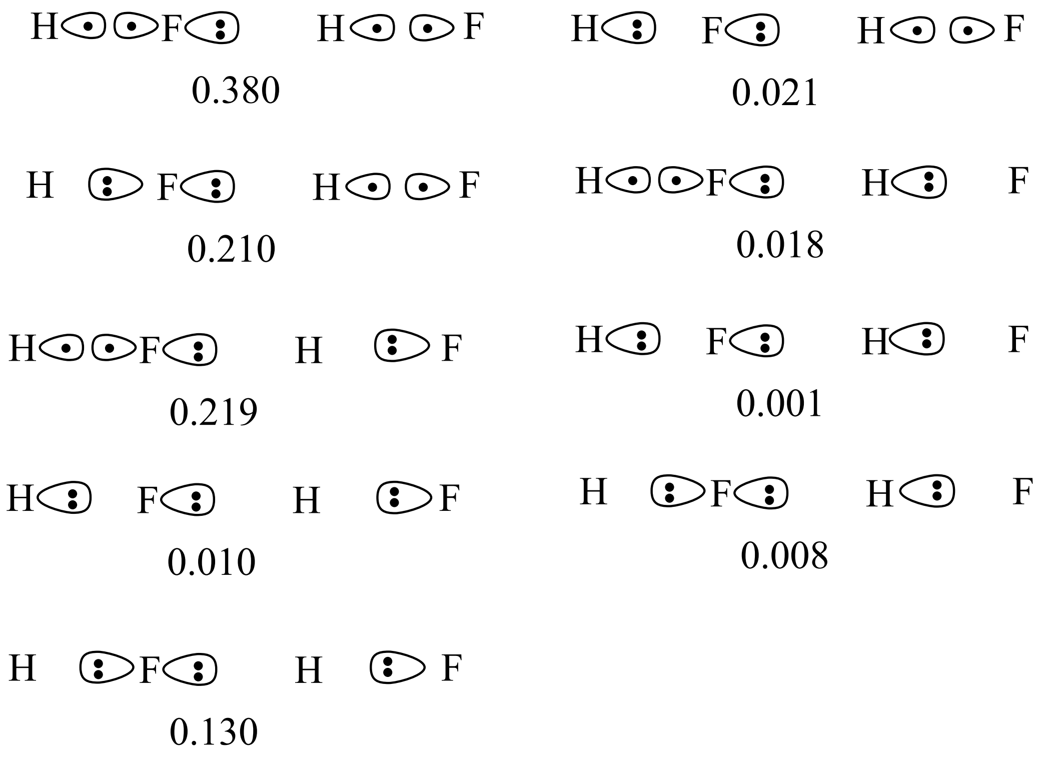

S5. Cartesian coordinates (in Å) from CCSD(T)/cc-pVTZ optimized structures.
All calculations were done using Gaussian 16 program[1]

Optimal distance				10 Å distance			
H_2CO---H-NH_2							
1	-1.104617	-0.082472	-0.000014	1	5.841944	0.052273	-0.000270
7	-2.083614	0.186399	-0.000062	7	6.853449	0.125840	-0.000318
8	1.033632	-0.669719	0.000090	8	-4.131712	-0.673116	0.000201
1	-2.491238	-0.272141	-0.807421	1	7.164957	-0.400131	0.808916
1	-2.491213	-0.271958	0.807426	1	7.165182	-0.404740	-0.806452
6	1.495443	0.451530	-0.000054	6	-4.359353	0.515306	-0.000157
1	0.847341	1.342991	0.000747	1	-3.545716	1.259526	-0.000823
1	2.583305	0.627358	-0.000705	1	-5.390694	0.905286	0.000185
H_2O---H-OH							
1	-0.564036	0.000337	0.041692	1	-4.589037	0.081354	0.009503
8	-1.525833	0.000892	0.117018	8	-5.548361	0.098851	0.011554
8	1.386211	-0.000791	-0.111047	8	5.409277	-0.101004	-0.011864
1	1.751248	0.760272	0.347376	1	5.745426	0.468021	-0.706901
1	1.751146	-0.755324	0.358126	1	5.745351	0.291496	0.796133
1	-1.821382	-0.006100	-0.794959	1	-5.789070	-0.823647	-0.096253
H_2N---H-CF_3							
1	0.558580	-0.000184	-0.000228	1	-1.190761	0.000008	-0.000044
6	-0.528507	-0.000082	-0.000089	6	-2.277322	0.000015	-0.000053

7	2.860894	-0.000400	-0.000524	7	8.809239	-0.000052	0.000033
9	-1.006823	-0.232728	-1.228258	9	-2.754605	-0.097561	1.242144
9	-1.006520	-0.947337	0.815742	9	-2.754664	1.124568	-0.536570
9	-1.006201	1.180154	0.412589	9	-2.754650	-1.027025	-0.705527
1	3.254040	0.767631	-0.533892	1	9.188041	0.073477	-0.936039
1	3.249665	0.081073	0.932836	1	9.188235	0.774013	0.531382
1	3.258393	-0.846039	-0.395173	1	9.189020	-0.847068	0.404359
HF---H-F							
1	0.041158	0.508261	0.000000	1	0.041011	4.607763	0.000000
9	0.041158	1.430570	0.000000	9	0.041011	5.525029	0.000000
9	0.041158	-1.297128	0.000000	9	0.041011	-5.392237	0.000000
1	-0.781996	-1.709247	0.000000	1	-0.779215	-5.802891	0.000000
H_3N---H-F							
1	0.494852	-0.000028	-0.000090	1	-4.644474	0.000010	-0.000028
9	1.438222	-0.000020	-0.000055	9	-5.561701	0.000005	-0.000013
7	-1.229322	-0.000044	-0.000153	7	5.355526	0.000066	-0.000197
1	-1.609724	-0.035995	0.938794	1	5.735542	-0.003788	0.938529
1	-1.611448	0.830957	-0.437254	1	5.737281	-0.810876	-0.471635
1	-1.612428	-0.794449	-0.499883	1	5.738280	0.814147	-0.465374
$(NC\text{--}H\text{-}CN)^-$							
1	0.000140	-0.371448	0.000000	1	-4.276153	0.000068	0.000009
6	0.000140	-1.498189	0.000000	6	-5.343599	0.000072	0.000004
6	0.000140	1.542100	0.000000	6	5.723847	0.000031	0.000049
7	0.000280	-2.662347	0.000008	7	-6.503631	-0.000062	-0.000019
7	0.000216	2.721462	-0.000067	7	6.907082	-0.000036	-0.000028
$(CN\text{---}H\text{-}NC)^-$							
1	0.000121	-0.103537	0.000000	1	-4.271878	0.000055	-0.000141
7	0.000121	-1.233271	0.000000	7	-5.269041	0.000086	-0.000140
7	0.000121	1.356513	0.000000	7	5.728122	-0.000252	-0.000151
6	0.000010	-2.408044	-0.000152	6	-6.443911	0.000196	0.000034
6	-0.000155	2.536357	-0.000477	6	6.911389	-0.000019	0.000331
$(HO\text{---}H\text{-}OH)^-$							
1	-0.000108	0.000062	0.069454	1	4.281034	-0.056575	-0.068504
8	-1.212740	-0.085261	0.069514	8	5.242669	-0.069716	-0.068242
8	1.212660	0.085395	0.069394	8	-5.718032	0.080064	-0.071225
1	-1.430068	0.578149	-0.590936	1	5.421747	0.589495	0.608053
1	1.430814	-0.579285	-0.589782	1	-5.899872	-0.615705	0.576185
$(F\text{--}H\text{-}F)^-$							
1	0.000240	-0.000805	0.000000	1	0.000000	0.000000	-4.302053
9	-0.000013	1.134586	0.000000	9	0.000000	0.000000	-5.219941
9	0.000497	-1.135124	0.000002	9	0.000000	0.000000	5.697947

References

(1) Gaussian 16, Revision B.01, Frisch, M. J.; Trucks, G. W.; Schlegel, H. B.; Scuseria, G. E.; Robb, M. A.; Cheeseman, J. R.; Scalmani, G.; Barone, V.; Petersson, G. A.; Nakatsuji, H.; Li, X.; Caricato, M.; Marenich, A. V.; Bloino, J.; Janesko, B. G.; Gomperts, R.; Mennucci, B.; Hratchian, H. P.; Ortiz, J. V.; Izmaylov, A. F.; Sonnenberg, J. L.; Williams-Young, D.; Ding,

F.; Lipparini, F.; Egidi, F.; Goings, J.; Peng, B.; Petrone, A.; Henderson, T.; Ranasinghe, D.; Zakrzewski, V. G.; Gao, J.; Rega, N.; Zheng, G.; Liang, W.; Hada, M.; Ehara, M.; Toyota, K.; Fukuda, R.; Hasegawa, J.; Ishida, M.; Nakajima, T.; Honda, Y.; Kitao, O.; Nakai, H.; Vreven, T.; Throssell, K.; Montgomery, J. A., Jr.; Peralta, J. E.; Ogliaro, F.; Bearpark, M. J.; Heyd, J. J.; Brothers, E. N.; Kudin, K. N.; Staroverov, V. N.; Keith, T. A.; Kobayashi, R.; Normand, J.; Raghavachari, K.; Rendell, A. P.; Burant, J. C.; Iyengar, S. S.; Tomasi, J.; Cossi, M.; Millam, J. M.; Klene, M.; Adamo, C.; Cammi, R.; Ochterski, J. W.; Martin, R. L.; Morokuma, K.; Farkas, O.; Foresman, J. B.; Fox, D. J. Gaussian, Inc., Wallingford CT, 2016.
